# Supplementary material for: Observing Plasticity of the Auditory System: Volumetric Decreases Along with Increased Functional Connectivity in Aspiring Professional Musicians
Source: Cereb Cortex Commun. 2021 Feb 9;2(2):tgab008. doi: 10.1093/texcom/tgab008 (PMC8152844; doi:10.1093/texcom/tgab008)
Supplement: Pitch-Study_Supplementary_Material_tgab008 [file pitch-study_supplementary_material_tgab008.zip › Pitch-Study_Supplementary_Material_tgab008.pdf]

Supplementary material.

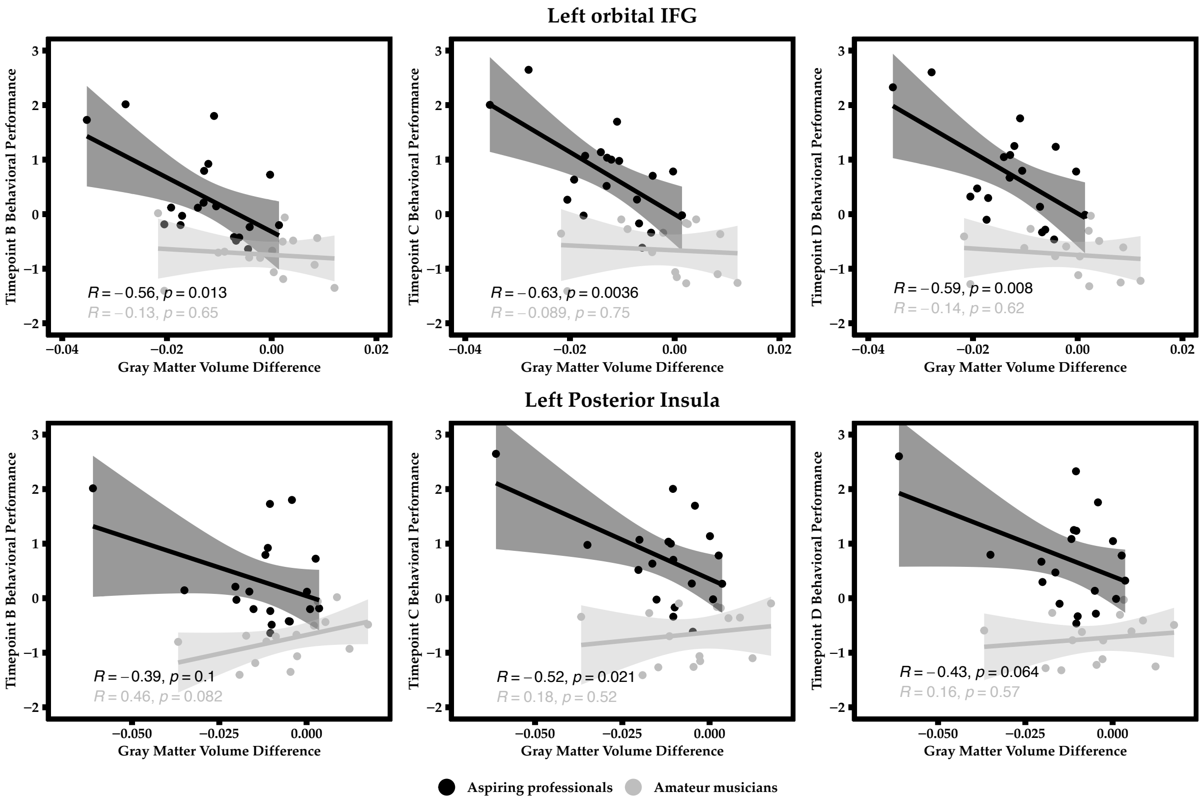

Figure S1. Correlations of gray-matter volume changes from measurement occasion B to D in left orbital inferior frontal gyrus (IFG) and left posterior insula inferior frontal orbital gyrus with behavioral performance at measurement occasions B, C, and D.

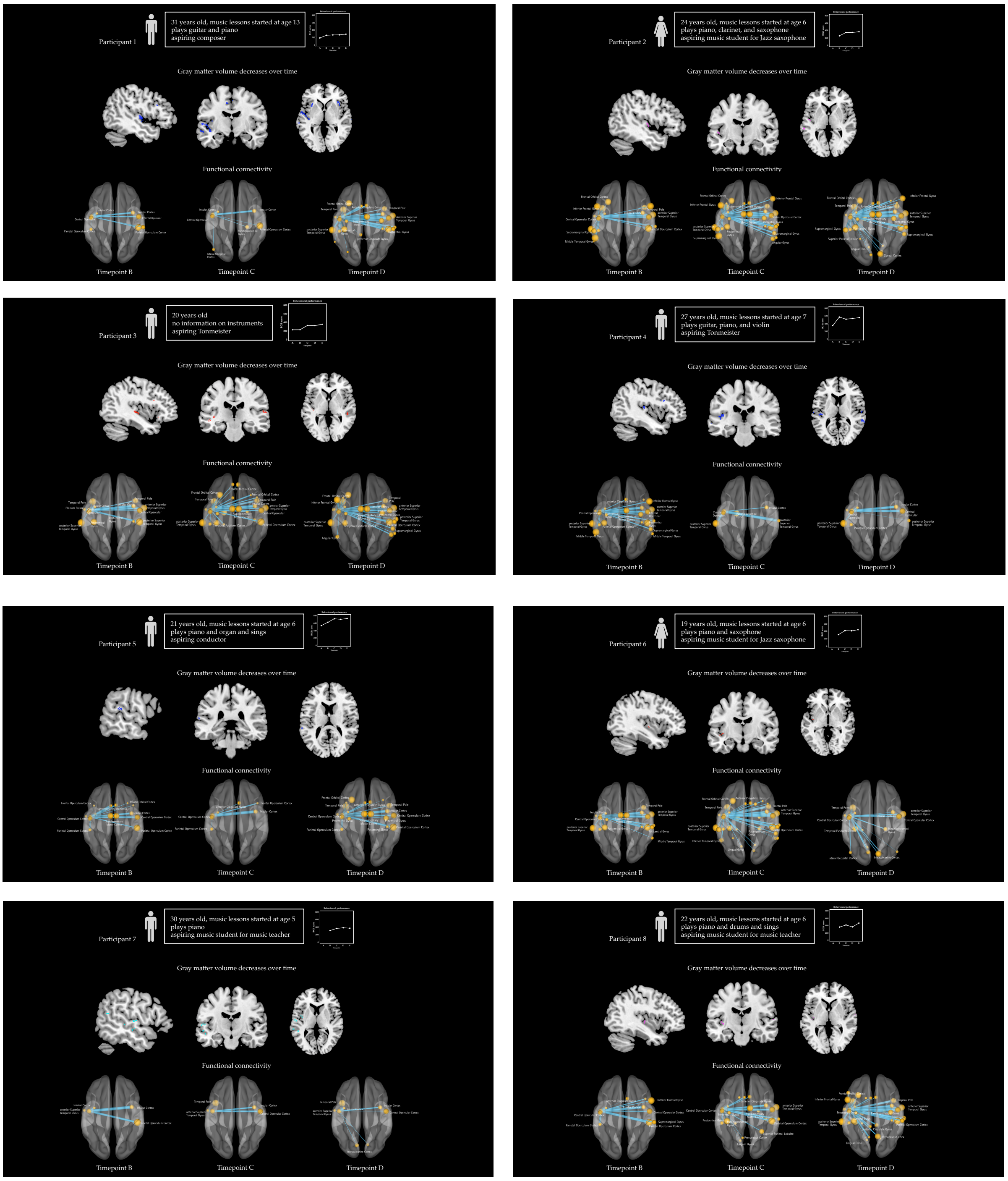

Figure S2. Individual-level data of 8 selected participants. The figure shows gray matter change over time, calculated with a linear regression over all images available for one subject ($p < .005$, corrected for non-stationary smoothness, cluster-size > 10 voxel), as well as plots on the functional connectivity at each time point for that participant (correlations of $r > 0.6$ are shown), along with demographical information, and their behavioral performance over time.

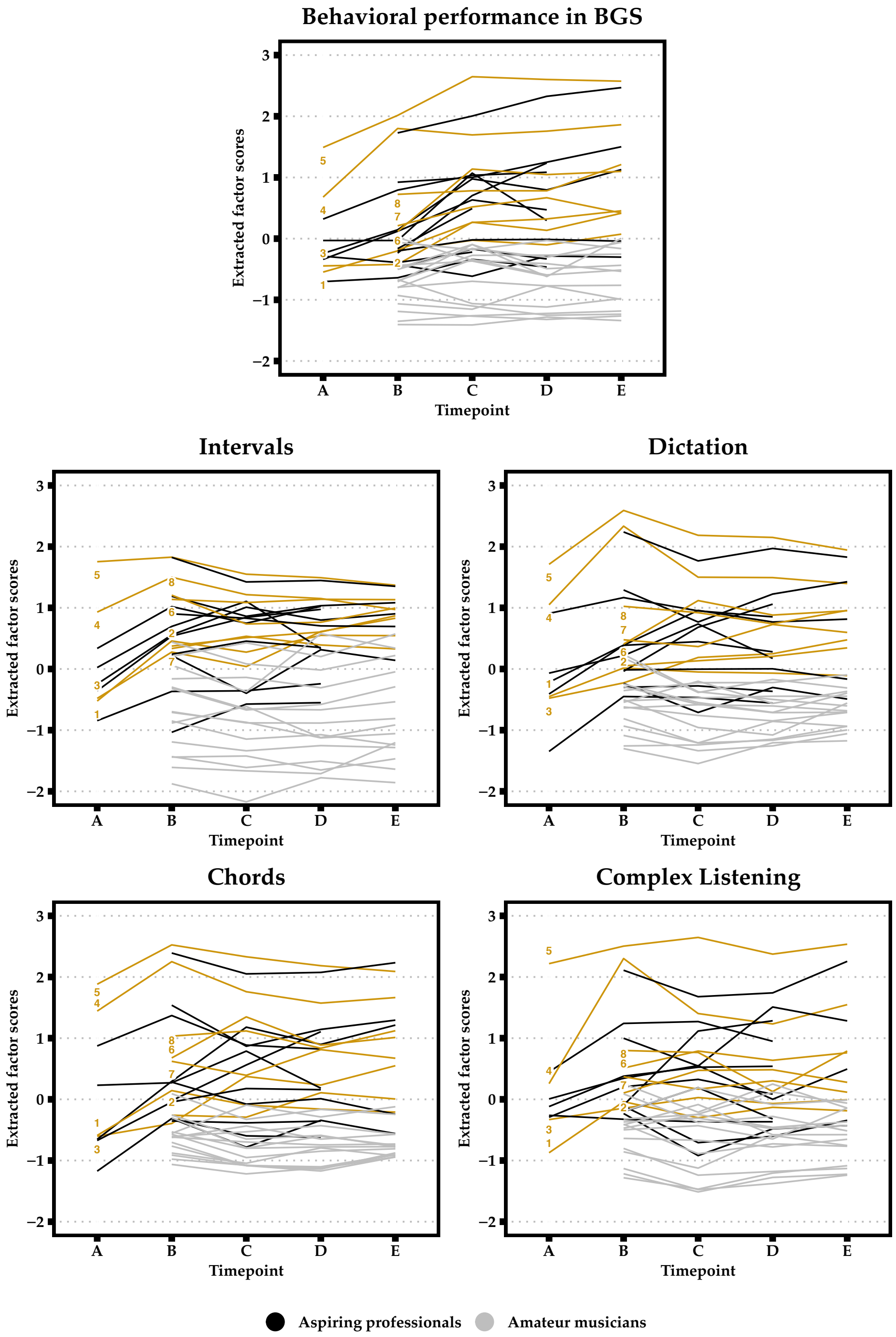

Figure S3. Line plots of individuals' time courses of the overall Berlin Gehoerbildung Scale (BGS) score representing music expertise, and the four factors representing sub-domains (Intervals, Dictation, Chords, and Complex Listening). Selected participants portrayed in Figure S2 (participants 1-8, all belonging to the group of aspiring professionals) are displayed in dark yellow lines here, to further illustrate their behavioral performance.
